# Climate and vegetation structure shape ant communities along elevational gradients on the Colorado Plateau

**DOI:** 10.1002/ece3.6538

**Published:** 2020-07-07

**Authors:** Derek A. Uhey, Richard W. Hofstetter, Michael Remke, Sneha Vissa, Karen A. Haubensak

**Affiliations:** ^1^ School of Forestry Northern Arizona University Flagstaff Arizona USA; ^2^ Mountain Studies Institute Durango Colorado USA; ^3^ Department of Biological Sciences and Center for Ecosystem Science and Society Northern Arizona University Flagstaff Arizona USA

**Keywords:** abundance, arthropods, communities, precipitation, primary productivity, richness, structural equation model, temperature

## Abstract

Terrestrial animal communities are largely shaped by vegetation and climate. With climate also shaping vegetation, can we attribute animal patterns solely to climate? Our study observes ant community changes along climatic gradients (i.e., elevational gradients) within different habitat types (i.e., open and forest) on the Colorado Plateau in the southwestern United States. We sampled ants and vegetation along two elevational gradients spanning 1,132 m with average annual temperature and precipitation differences of 5.7°C and 645mm, respectively. We used regression analyses and structural equation modeling to compare the explanatory powers and effect sizes of climate and vegetation variables on ants. Climate variables had the strongest correlations and the largest effect sizes on ant communities, while vegetation composition, richness, and primary productivity had relatively small effects. Precipitation was the strongest predictor for most ant community metrics. Ant richness and abundance had a negative relationship with precipitation in forested habitats, and positive in open habitats. Our results show strong direct climate effects on ants with little or no effects of vegetation composition or primary productivity, but contrasting patterns between vegetation type (i.e., forested vs. open) with precipitation. This indicates vegetation structure can modulate climate responses of ant communities. Our study demonstrates climate‐animal relationships may vary among vegetation types which can impact both findings from elevational studies and how communities will react to changes in climate.

## Graphical abstract

Animal communities are largely shaped by climate and vegetation. Comparing these effects on ant communities along an arid elevational gradient, we show that climate effects outweigh vegetation.

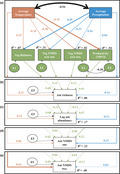

## 1 INTRODUCTION

A longstanding goal of biogeographers has been to understand the environmental factors that shape the distributions of biological communities (Hortal, Lobo, & Jiménez‐Valverde, 2012; Pianka, 1966; Rosenzweig, 1995). Climate is a key driver in shaping biological communities, correlating strongly with primary productivity (Roy, Mooney, & Saugier, 2001). For higher (consumer) trophic levels, climate effects occur directly through physiological effects or indirectly through changes to plant communities and food resources. However, the relative effects of climate and plants in shaping higher trophic communities remain vague, especially when considering biogeographical distributions across climatic gradients (Hortal et al., 2012; Wisz et al., 2013).

Climate affects organisms largely through precipitation and temperature (Pörtner & Farrell, 2008). Temperature predictably declines an average of 6°C with every 1,000 m gained in elevation (Barry, 1992), and elevational gradients are commonly used to study climate effects on terrestrial communities (Rahbek, 1995). As temperature decreases, so do metabolic processes which may decrease niche space and speciation rates (i.e., metabolic theory, Brown, Gillooly, Allen, Savage, & West, 2004). This produces monotonic declines in richness with increased elevation for most taxa (Rahbek, 1995). Unlike temperature, precipitation may increase or decrease with elevation depending on local climate. Precipitation, especially in arid ecosystems where it is a limited resource, acts as a trophic currency influencing species patterns and interactions (Allen, McCluney, Elser, & Sabo, 2014; McCluney et al., 2012). Studies in arid regions where precipitation increases with elevation observe variable richness patterns (reviewed by Szewczyk & McCain, 2016), even increases in richness with elevation (Sanders, Moss, & Wagner, 2003). Incorporating precipitation with temperature refines elevational predictions (i.e., the elevational climate model hypothesis, McCain, 2007), and suggests that optimal climate ranges exist on elevational gradients where high precipitation and temperatures coincide. The location along a given elevational gradient where this optimal climate occurs is likely to have the highest productivity and diversity. Most elevational studies on animal communities and climate attempt to frame results within one of the aforementioned hypotheses (Szewczyk & McCain, 2016). However, the above hypotheses fail to separate vegetation and climate effects on higher trophic levels.

Our study's biophysical setting is the Colorado Plateau, a large arid region of the southwestern United States interspersed with mountains that form significant elevational gradients. This region contains life zones (i.e., biological communities found in similar elevations/latitudes) representative of most of western North America (Merriam, 1898) within a localized species pool. Several of these life zones include both forest and open habitats, which differ enormously in vegetation structure, composition, and productivity. This provides an opportunity to compare vegetation effects on animal communities within the same climate zone. We focus on ant (Hymenoptera: Formicidae) communities, which are dominant in most terrestrial food webs (Hölldobler & Wilson, 1990). Ants are commonly used as indicators of general ecological responses to disturbance (Andersen & Majer, 2004) and may be indicative of environmental stress (Tiede et al., 2017).

Elevational gradients patterns of ant richness are well‐documented: richness usually declines monotonically with elevation, but can peak at mid‐elevations (reviewed by Szewczyk & McCain, 2016). Temperature drives ant metabolism (Brown et al., 2004), foraging rates (Vogt, Smith, Grantham, & Wright, 2003), and inter‐ and intra‐specific competition (Cerdá, Retana, & Manzaneda, 1998). Ant species richness positively correlates with temperature at global scales (Gibb et al., 2015; Jenkins et al., 2011); and few ants exist at high elevations or extreme latitudes. Precipitation also shapes ant communities (Weiser et al., 2010), especially in arid systems where ant diversity increases with moisture (Supriya, Moreau, Sam, & Price, 2019). Precipitation is vital for colony founding and larval/pupal development (Johnson, 1998). On arid gradients, low‐elevations are extremely dry, while high‐elevations are cold, which can cause mid‐elevational peaks in ant diversity (Nowrouzi et al., 2016; Szewczyk & McCain, 2019). However, attributing elevational patterns of ants to climate variables are fraught with complexities because of covariation of vegetative communities along elevational gradients.

Ants are either directly or indirectly dependent on vegetation for food and often for nesting space (Hölldobler & Wilson, 1990). Vegetation structure controls levels of insolation creating microclimates, modulating environmental stress (Bolger, Kenny, & Arroyo, 2013). This is best demonstrated between open and forested habitats, where ant communities vary markedly (Lassau & Hochuli, 2004). Yet many elevational studies designed to assess climate effects are carried out in different vegetation types (e.g., Nakamura et al., 2016). With increased elevation, ants nest in more insolated locations (Plowman et al., 2020) and along elevational gradients ant community composition can closely follow dominant vegetation (e.g., Andersen, 1997). Lasmar et al. (2020) found conflicting patterns of ant diversity between open and forested habitats along elevational gradients in Brazil. Such variability indicates that disentangling diversity patterns from climate and vegetation may be important.

Here, we investigate the relative effects of climate and vegetation on ant communities across two elevational gradients. We asked: (a) What climate and vegetation variables best explain patterns in ant richness, abundance, and composition? (b) Do these patterns change in different habitat types (forest vs. open)? and (c) What are the relative and indirect effects of climate and vegetation on ant communities? We use structural equation modeling (SEM) to the compare multiple casual paths including indirect effects, allowing for stronger causal inference from nonexperimental data. In this arid system, we expected precipitation to have the strongest effects on ants.

## 2 METHODS

### 2.1 Study sites and design

**FIGURE 1:**
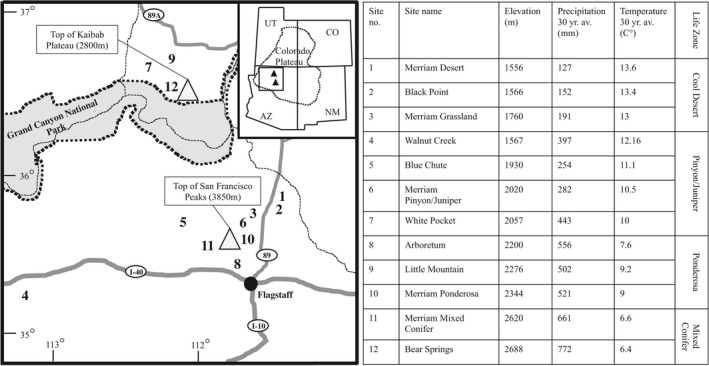
Location and climate/biome descriptions of elevational study sites in northern Arizona, on the Colorado Plateau region in the southwestern United States. Sites are coded by shape and life zone by shape fills

To examine variation in richness, abundance, and composition of ant communities across habitat types, we selected 12 sites across two elevational gradients located on the Colorado Plateau with considerable climate differences (Figure 1, Table S1). Both gradients encompass the following life zones spanning 1,556–2,688 m above sea level: cool desert, pinyon/juniper, ponderosa pine, and mixed conifer. From the lowest to highest elevation sites, average annual temperature decreases from 13.6 to 6.7°C and average precipitation increases from 127 to 772 mm/year, resulting in positive correlations of productivity with elevation (detailed site descriptions ‐ Smith, Higgins, Burton, and Cobb, (2015) and https://www.sega.nau.edu/). Productivity and vegetation composition were distinctly different within seven of our 12 sites (located in pinyon/juniper and ponderosa life zones), with structurally complex and high‐biomass forests adjacent to open (meadow) habitats. In these seven sites (all located in either the pinyon/juniper or ponderosa life zones), plots were paired as forested or open habitats. Single plots were established at the three cool desert sites which had only open habitat available, and two mixed‐conifer sites which had only forest habitat available, leading to a total of 19 plots (10 open and 9 forested). Plots were 30 × 30 m (900 m^2^) in size and located at least 100 m from disturbed areas such as roads. Within each 900 m^2^ plot, five subplots were used for ant community sampling and vegetation cover measurements. These five (1 m^2^) subplots were located as follows: One in the center and four located halfway between the center subplot and the corners of the large plot such that all subplots were separated at least 10 m from one another and from the edge of the 900 m^2^ plot (Figure S1).

### 2.2 Ant community

A pitfall trap (Thomas & Sleeper, 1977) was dug in each subplot, a reliable method for sampling different ground‐dwelling arthropod communities with equal sampling intensity (Andersen, Hoffmann, Müller, & Griffiths, 2002). Pit traps consisted of a long borosilicate glass tube measuring 32 mm diameter and 200 mm length filled with 100 mm of propylene glycol fitted within PVC sleeves with a rain cover (Higgins, Cobb, Sommer, Delph, & Brantley, 2014). Ant communities were sampled for 7‐day periods during the dry season (9–16 June 2015) on 89 subplots and during the rainy season (6–13 August 2015) on the same subplots, minus 14 which were flooded (Table S1). Pit trap samples were sorted, and voucher specimens of each species and site occurrence were identified with specialist help and deposited at the Colorado Museum of Arthropod Biodiversity at Northern Arizona University (Table S2), yielding richness and abundance measures for each trap. Species accumulation curves show most sites approach an asymptote in for sampling (Figure S2).

### 2.3 Vegetation, weather, and climate measurements

Percent cover of herbaceous vegetation was estimated using a point intercept method with 25 points on a grid pattern within a 1 m^2^ circular plot centered over the pit trap at each subplot (methods modified from Godínez‐Alvarez, Herrick, Mattocks, Toledo, & Van Zee, 2009). Vegetation was identified to species for richness and composition. To estimate productivity, we used normalized difference vegetation index (NDVI, Pettorelli et al., 2005) calculated from satellite imagery at the 250 m^2^ resolution for each site and sampling date (Dimiceli et al., 2015). Weather was measured either by on‐site weather stations or temperature data loggers at each plot. Climatic factors were established for each site using 30‐year averages of annual precipitation and temperature (Table S2). All climate data were extracted using PRISM Climate Group, Oregon State University (PRISM Climate Group, Oregon State University, http://prism.oregonstate.edu, created 03 May 2015). PRISM data were downloaded at a spatial resolution of 800 meters and extracted using R version 3.2.3 (2015‐12‐10) © 2015 The r Foundation for Statistical Computing Platform: x86_64‐apple‐darwin13.4.0 (64‐bit) and the package “raster” version 2.5‐2 based on observed latitude and longitude of each site.

### 2.4 Analysis

To represent ant and vegetation composition, nonmetric multidimensional scaling (NMDS) ordination (McCune, Grace, & Urban, 2002) on sites was used for both wet and dry sampling periods (i.e., pit traps were averaged for each site/sampling period). Nonmetric multidimensional scaling (NMDS) was required as a result of the abundance of zeros in the data, using a Bray‐Curtis distance metric (2D stress (S_R_) = 17.06) (McCune et al., 2002). The two axes that explained the most variation in ant communities were chosen, after which the ordination was rotated so that elevation aligned with axis one. The same ordination procedure was performed on the vegetation community (measured as percent cover for each plant species, 2D stress (S_R_) = 12.16), which then represented vegetation composition in regression and ordination analyses (e.g., vegetation composition one and two).

Site averages of climate (average annual temperature and precipitation), weather (precipitation and temperature during sampling), and vegetation (NDVI, composition, and richness) were used as explanatory variables for ant community metrics (richness, abundance, and composition). We used average (opposed to total) richness because of uneven sampling driven by a flooding event. However, average richness correlated with total richness (*r* = .739, *R* ^2^ = .518) in this case. All variables were checked for normality via Shapiro‐Wilk tests and transformed when necessary. To test explanatory variables of climate and vegetation, stepwise multiple regressions with backwards elimination (*p* = .05) were used on ant richness and abundance. Ordinations were used to examine ant composition relatedness between life zones and habitats, and to test correlations of climate, weather, and vegetation. To determine if the visual separation of groups (life zone/habitats) was significant between ant communities, a one‐factor permutational multivariate analysis of variance (perMANOVA) (Anderson & Walsh, 2013) was performed on the ant community with life zone as a fixed effect, followed by pairwise comparisons (Table S3). Normality checks, PerMANOVAs, and regression analyses were conducted in r version 3.5.0 using the “vegan” package (Oksanen et al., 2019). Indicator analyses and ordinations were run on pcord version 6.08 (McCune & Mefford, 2011).

### 2.5 Structural Equation Modeling (SEM)

**FIGURE 2:**
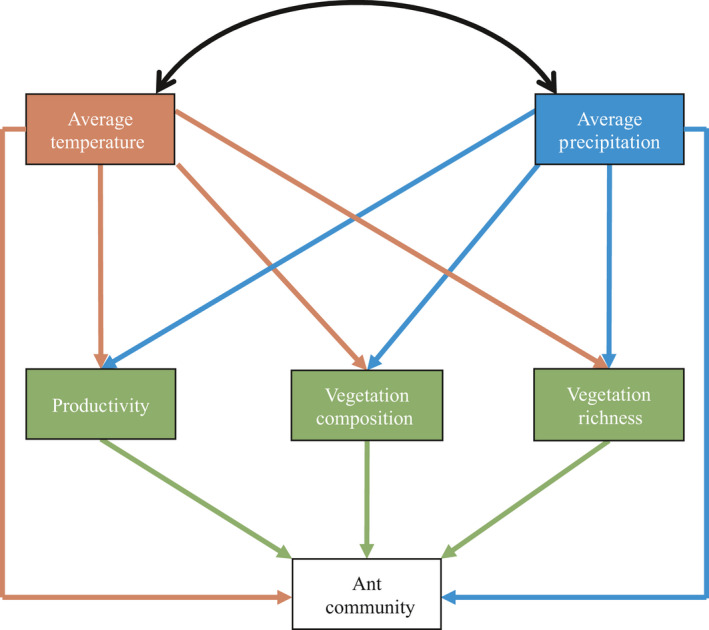
A priori model of climate (measured by average annual temperature and precipitation) and vegetation (measured by primary productivity, composition, and richness) effects on ant communities. Variables are color coded to match causal pathways. Climate effects on ants are both direct and indirect through vegetation. The double‐headed black arrow indicates the known correlation between temperature and precipitation. Different measurement models were made for each ant community metric (richness, abundance, and composition)

To test the relative direct and indirect effects of climate and vegetation on the ant community, structural equation modeling (SEM, Grace, 2006) was used. An a priori model of our system was first created based on the simple assumptions that vegetation influences ants, and climate influences both vegetation and ants. A measurement model incorporating climate and vegetation data was developed and suggested by literature (Figure 2). Vegetation composition was represented as two separate variables to include both axes of the NDMS ordination. A separate model was tested for each ant community measure: richness, abundance, composition one (NMDS axis one), and composition two (NMDS axis two). To achieve necessary sample sizes for SEM, analysis was conducted with plot‐level data instead of site averages. This approach results in pseudo‐replicates for average annual temperature and precipitation, and NDVI (Schank & Koehnle, 2009). The model was formulated in amos 5.0 (2003 spss Inc.). For each endogenous variable, error terms are assigned to represent unexplained variance. The model suggested a correlation between the error terms of vegetation NDMS axis two and productivity which was added post hoc. The final models were evaluated with Joreskog's goodness‐of‐fit (GIF) and *X* ^2^ tests. Contrary to most tests, a high *p* value indicates a good probability that a model fits the data and is thus desired. GIF > 0.95 are considered a good fit (Grace, 2006). Effect sizes are estimated with standardized path coefficients, analogous to weighted regressions, which show effect direction (positive or negative) and effect size (the further the value is from 0, the stronger the effect).

## 3 RESULTS

A total of 5,300 ants were collected from the two sampling periods in 2015, with 36 identifiable taxa [34 species including one morpho‐species, two species complexes, and one genus with multiple unidentified species (*Myrmica*)]. One species (*Monomorium cyaneum*) and one species group (*Solenopsis fugax* group) were ubiquitous (Table S4). Richness and abundance were both highest at mid‐elevation sites (Figure S3). In general, climate variables and vegetation composition were strong predictors for ant community metrics, while vegetation richness, NDVI, and weather (real‐time measures of temperature and precipitation) variables were not (Tables 1 and 2). All ant community metrics showed relationships with annual average precipitation. Ant richness and abundance were associated with vegetation composition, while ant composition was closely tied to annual average temperature (Tables 1 and 2). One part of ant composition (NMDS axis one) responded to precipitation, while another (NMDS axis two) responded to temperature (Figure 4), each axis was associated with a different suite of ant species (Table S4). For example, axis one correlated with *Tapinoma sessile* (*r* = .3), *Dorymyrmex insanus* (*r* = −.32), *Forelius mccooki* (*r* = −.31), *F. pruinosus* (*r* = −.49), *Pheidole bicarinata* (*r* = −.35), and *Pheidole ceres* (*r* = −.43), while axis two correlated with *Formica aserva* (*r* = .42), *Formica neogagates* (*r* = .31), *Camponotus modoc* (*r* = −.44), *Pogonomyrmex rugosus* (*r* = .38), *Monomorium cyaneum* (*r* = −.30), and *Crematogaster punctulata* (*r* = −.42). Therefore, the patterns of NMDS axis one and two are indicative of two different species sets.

**TABLE 1:** Results of backwards stepwise multiple regressions for average ant richness and (log) abundance in open and forest elevational sites, as a function of climate and vegetation variables

Dependent variable	Independent variable(s)	*r*	*β*	*p*	%var	Σ%var
Open ant richness	Av. ann. precipitation	0.298	0.520	.039	15.496	
	Veg NMDS axis one	0.333	0.540	.032	17.982	33.478
Open ant abundance	Av. ann. precipitation	0.427	0.456	.040	19.471	
	Veg NMDS axis two	−0.393	−0.445	.045	17.489	36.960
Forest ant richness	Av. ann. precipitation	−0.544	−0.480	.007	26.112	
	Veg NMDS axis two	−0.533	−0.331	.001	17.642	43.754
Forest ant abundance	Av. ann. precipitation	−0.593	−0.776	<.001	46.017	46.017

Note

*r*, Pearson's correlation coefficient; *β*, standardized partial regression coefficient; %var, contribution of each independent variable to the prediction of the dependent variable (100 × *β* × *r*); and Σ%var, accumulated percentage of variance explained.

**TABLE 2:** Direct and indirect vegetation mediated (summed across all vegetation metrics) standardized effects of average annual precipitation and temperature on ant community metrics

Response	Precipitation effect (standardized estimates)		Temperature effect (standardized estimates)	
	Direct	Σ(veg mediated)	Direct	Σ(veg mediated)
Av. ant richness	0.16	−0.0391	0.21	−0.078
Av. ant abundance	−0.31	−0.0025	0.22	−0.002
Ant NMDS axis one	0.33	0.0268	0.06	−0.179
Ant NMDS axis two	0.03	0.0367	0.64	0.0955

### 3.1 Ant composition varies by life zone, abundance/richness varies by habitat

**FIGURE 3:**
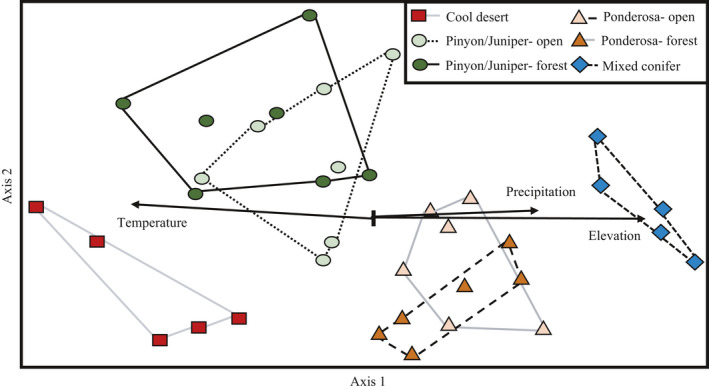
Nonmetric multidimensional (NMDS) ordination of ant taxonomic groups in four life zones, as well as two habitat types (open and forest) within pinyon‐juniper and ponderosa life zones. Site that is closer together is more similar in ant composition. Ordination was rotated to align axis one with elevation (*R* ^2^ = .853), shown as an arrowed line. Ant taxa cluster into separate life zones as shown by convex hulls, while ant taxa from forest and open microhabitats within the same life zone do not form separate clusters. Significance of these separations was tested via pairwise perMANOVA (Table S5). Significant correlations of average annual air temperature (*R* ^2^ = −.0.607), average annual precipitation (*R* ^2^ = .643), and elevation are shown as solid lines. Each axis is associated with a different set of species (Table S4)

Ant composition grouped by life zone, while ant composition in open and forest habitats overlapped (Figure 3, Table S5). Indicator species were found in each life zone and habitat combination (Table S5), but overall ant abundance and richness were not significantly different between life zones and/or habitats (Figure S4). Patterns in ant richness and abundance only became significant after forest and open sites were considered separately. Variance in ant richness and abundance of both open and forest sites was best explained by precipitation and vegetation composition. Precipitation was positively correlated to forest ant richness and abundance, and negatively correlated to open ants (Table 1).

### 3.2 Climate has strong direct effects on ants relative to vegetation

**FIGURE 4:**
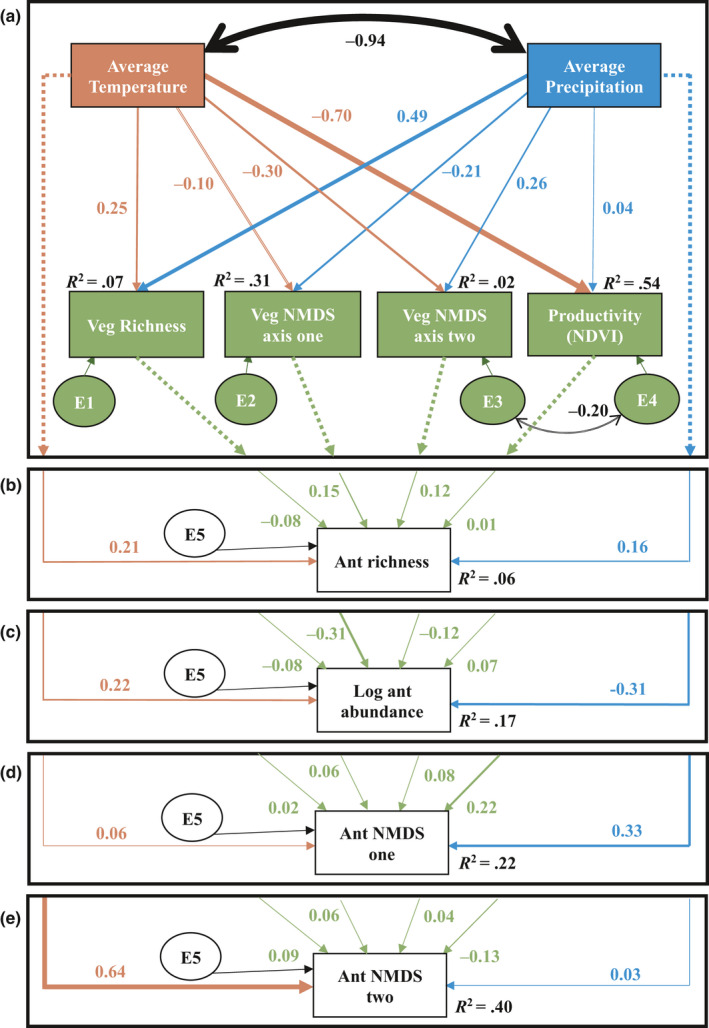
Structural equation model of ant community responses to climate and vegetation, demonstrating differential effects of climate and vegetation on ant community metrics. Panel (a) displays the higher model structure of climate effects on vegetation, with subsequent panels showing climate and vegetation effects on four ant community metrics: average ant richness (b), log average ant abundance (c), and two axes of a NMDS ordination representing composition (d‐e) with each axis associated with a different suite of species (Table S4). *R^2^* and error terms are indicated next to endogenous variables. Error terms, representing unexplained variance, are shown as circles labeled E1‐5. Arrows indicate unidirectional causal relationships. Proportions next to arrows indicate standardized path coefficients (equivalent to correlation coefficients) with arrow width sized proportionally. Broken lined arrows in the top panel indicate paths that change as different ant community metrics are considered. All four models fit the data equally well

Climate variables had strong direct effects on both vegetation and ants (Figure 4), and the data fit our SEM model well (*χ* ^2^_6_ = 6.83, *p* = .234, RM* r* = .018, RMSEA‐0.047, GFI = 0.987, CFI = 0.997, AIC = 52.83, BIC = 127.127).Vegetation, and vegetation‐mediated effects of climate, had weak effects on ants compared with direct climate effects (Table 2). Vegetation composition was an exception in the case of ant abundance (standardized path coefficient, −0.31). There were differences in how predictor climate‐ and vegetation‐predictor variables affected ants depending on which community metric was used. Precipitation had notably strong effects on average ant abundance (−0.31) and ant NMDS axis one (0.33), while temperature was the best predictor for ant NMDS axis two (0.64). Several moderate effects on ants were also evident: temperature on ant richness (0.21) and abundance (0.22) and primary productivity on ant NMDS axis one (0.22).

## 4 DISCUSSION

Climate had strong direct effects on ants with little or no effects of vegetation composition or primary productivity, but vegetation type (i.e., forested vs. open) had contrasting relationships with precipitation. This indicates the influence of vegetation on ant communities along our elevational gradients is primarily through vegetative structure rather than plant composition or productivity. Our approach is correlative, and caution must be used when assigning causality. However, within our life zones and habitats, the model fit well, with strong explanatory variables for most ant community metrics. Our results are therefore indicative of how large‐scale climate and vegetation mechanisms shape animal communities along elevational gradients.

Hypothesized mechanisms shaping elevational distributions of animals are largely climate and geography based, mainly considering vegetation a by‐product (Szewczyk & McCain, 2016). Comparisons of vegetation and climate effects across elevations or latitudes usually find that climate is the strongest driver (Donoso, Johnston, & Kaspari, 2010; Sanders, Lessard, Fitzpatrick, & Dunn, 2007). Yet vegetation structure can modulate climate‐animal relationships (Carneiro, Mielke, Casagrande, & Fiedler, 2014; Lasmar et al., 2020), and similar patterns are well known for climate‐plant relationships (Michalet, Schöb, Lortie, Brooker, & Callaway, 2014). Stressful environments promote facilitation instead of competition in plants (i.e., the stress gradient hypothesis, Callaway et al., 2002). In forest plant communities, there is decreased competition and increased facilitation with increased elevation while in open communities show the reverse patterns (e.g., Sthultz, Gehring, & Whitham, 2007). Forest habitats retain moisture better, meaning higher/colder environments at high elevation are more stressful; while open sites are stressed by lack of precipitation at lower elevations (Michalet et al., 2014). This same climate stresses likely shape animal‐communities and seem to be behind our observed patterns.

Links between climate and ants are clear (Szewczyk & McCain, 2016), but effects of vegetative influences on ant communities are less so. Assumingly then, the interactive effects of climate and vegetation are even less clear. Ants rely on vegetation for resources such as food and shelter and in some instances ant species can be dependent on particular species of vegetation (Hölldobler & Wilson, 1990). Vegetation composition, richness, and productivity had small effects on ants, while vegetation structure (i.e., open vs. forested structure), which modulates climate, was the most influential aspect of vegetation. By offering ants more nesting opportunities and retaining more soil moisture than adjacent open habitats (Michalet et al., 2014), forests may buffer the effects of low precipitation on ants. Ants are adapted to certain nesting conditions and nests in open habitats must largely be constructed in soil (Lassau & Hochuli, 2004). Soil nesting success is highly contingent on soil moisture (Johnson, 1998), potentially explaining our positive relationship of ants with precipitation in open habitats. However, the negative relationship of ant richness and abundance with precipitation in forests indicates that ants in these habitats are not limited by precipitation. Rather, ants in these habitats may be affected by too much precipitation (i.e., flooding), or more likely limited by temperature which negatively correlates with precipitation. Along ours and most other temperate/arid gradients forests that exist at higher elevations and become too cold for most dominate ant species (Andersen, 1997).

Our results taken with others (e.g., Sanders et al., 2003; Szewczyk & McCain, 2016) suggest that lack of precipitation is just as limiting as cold temperatures for ants, especially in open habitats. Temperature had positive, moderate effects on ant richness and abundance, while precipitation had strong negative effects on ant abundance. Both temperature and precipitation had strong effects on ant composition which were positive or negative depending on ant species, suggestive of differences in physiological tolerance among species. Temperature, in general, has a positive relationship with ant richness and abundance (McCain, 2007) and favors thermophilic ant species which can become dominate leading to destabilization of communities (Diamond et al., 2016; Pelini et al., 2011; Stuble et al., 2013). Precipitation acts on ant communities as a trophic currency (reviewed by McCluney et al., 2012), but we know far less about it is effects on ant communities. Precipitation, taken with temperature and habitat type, is powerful explanatory variables for patterns in ant communities and distributions (Jenkins et al., 2011).

Communities can be measured by different metrics, and we chose three (richness, abundance, and composition) which all responded differently to climate and vegetation. Community metrics typically respond differently to environmental variables (e.g., Hillstorm and Lindroth, 2008) and are indicative of different ecological services (Winfree, Fox, Williams, Reilly, & Cariveau, 2015). Finding good predictors for ant community metrics can inform what future species assemblages and ecosystems will look like. Ant abundance and richness were closely associated with precipitation and vegetation composition, while ant composition was associated with temperature, precipitation, and vegetation richness. While most elevational studies test total richness, we choose instead to compare as many sites and habitats as possible with an averaged richness to ensure even comparisons from sampling differences caused by flooded traps and different habitat availability. Average richness correlated well with total richness and species accumulation curves give support that we sampled most species at our sites. However, our patterns based on average richness may be more reflective of abundant dominate species while under representing some rare species.

## 5 CONCLUSION

Understanding how climate and vegetation shape higher trophic communities and distributions continues to be a major challenge for biogeographers and ecologists. Our study demonstrates that climate‐animal relationships are strong, but may vary among vegetation types, which should be considered when sampling along elevational gradients. Documenting patterns along elevational gradients is increasingly important as climate changes. The Colorado Plateau and the entire southwestern United States is warming and drying (Polade, Pierce, Cayan, Gershunov, & Dettinger, 2014). Our results suggest this will drastically change ant communities in this region, potentially benefiting thermophilic species especially at higher elevations and limiting ant species in open habitats at lower elevations.

## CONFLICT OF INTEREST

None to declare.

## AUTHOR CONTRIBUTIONS

**Derek A. Uhey:** Conceptualization (equal); data curation (lead); formal analysis (lead); investigation (equal); methodology (equal); visualization (lead); writing – original draft (lead); writing – review and editing (lead). **Richard W. Hofstetter:** Conceptualization (equal); data curation (equal); funding acquisition (equal); investigation (equal); methodology (equal); project administration (equal); resources (equal); supervision (equal); writing – review and editing (equal). **Michael Remke:** Conceptualization (supporting); formal analysis (supporting); methodology (supporting); writing – review and editing (equal). **Sneha Vissa:** Visualization (equal); writing – original draft (equal); writing – review and editing (equal). **Karen A. Haubensak:** Conceptualization (equal); data curation (equal); funding acquisition (equal); investigation (equal); methodology (equal); project administration (equal); resources (equal); supervision (equal); writing – review and editing (equal).

## Supporting information

Appendix S1

## Data Availability

Data submitted to dryad: https://doi.org/10.5061/dryad.sbcc2fr3q.
