# Supplementary material for: Climate and vegetation structure shape ant communities along elevational gradients on the Colorado Plateau
Source: Ecol Evol. 2020 Jul 7;10(15):8313–22. doi: 10.1002/ece3.6538 (PMC7417256; doi:10.1002/ece3.6538)
Supplement: Supplementary file 1 — Appendix S1 [file ECE3-10-8313-s001.docx]

**Appendix S1**

**Title:** Climate and vegetation structure shape ant communities along elevational gradients on the Colorado Plateau

**Authors:** Uhey, D. A., Hofstetter, R. W., Remke, M., Vissa, S., & Haubensak, K. A.

*Derek A. Uhey (contact author), Richard W. Hofstetter, & Sneha Vissa, School of Forestry, Northern Arizona University, 200 E. Pine Knoll Dr., Flagstaff, AZ, 86011.*

*E-mail:* [*dau9@nau.edu*](mailto:dau9@nau.edu)

*Dr. Michael Remke, Mountain Studies Institute, 679 E. Second Ave., Durango, CO, 81301.*

*Dr. Karen A. Haubensak, Department of Biological Sciences and Center for Ecosystem Science and Society, Northern Arizona University, 617 N. Beaver Rd., Flagstaff, AZ, 86011.*


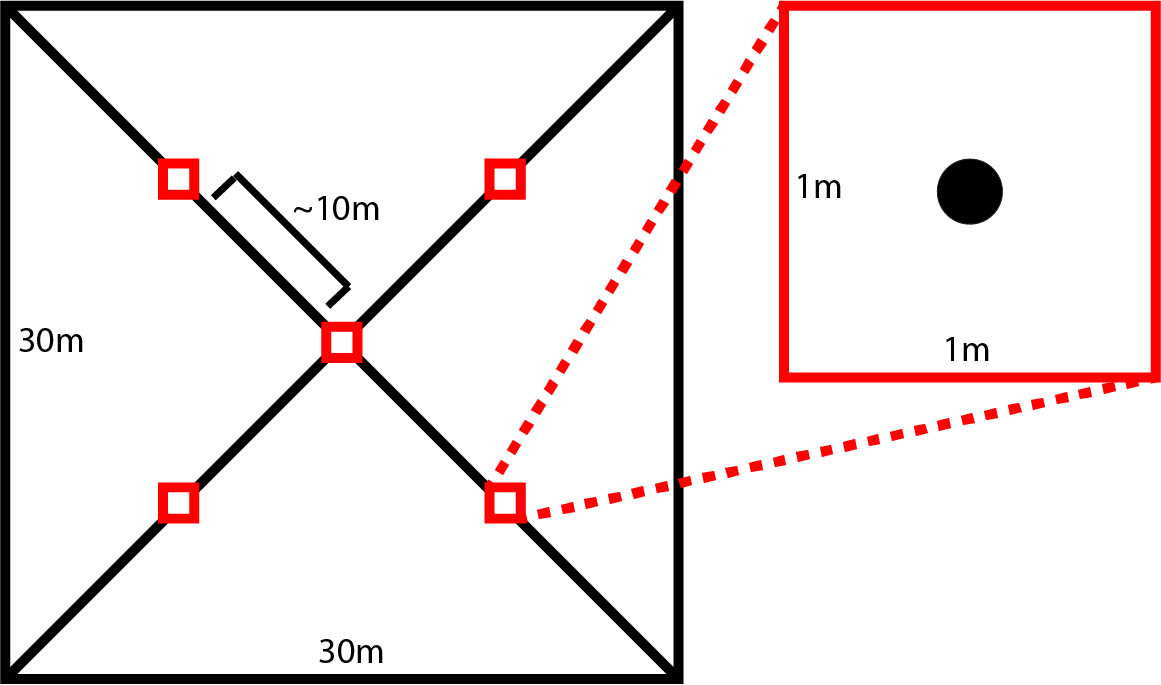


**Figure S1.** Plot design for elevational study. 1m^^2^ subplots are denoted in red and are positioned ~10m from each other and the edge of the plot. A pit trap was placed in the center of each subplot.


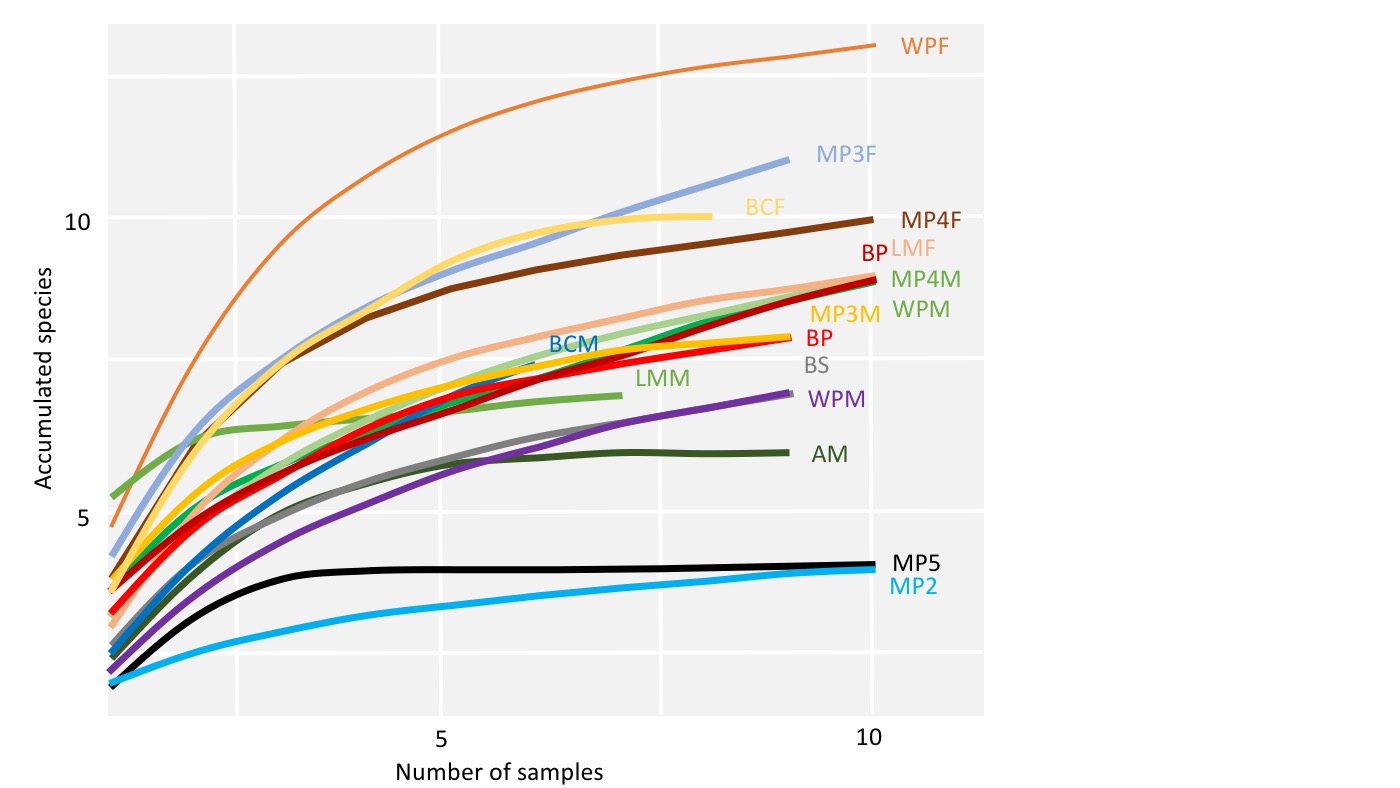


**Figure S2.** Species accumulation curves for elevational sites/habitat treatments (abbreviations in Supp. Table 1). Each sample represents a pitfall trap catch over a single sampling period.


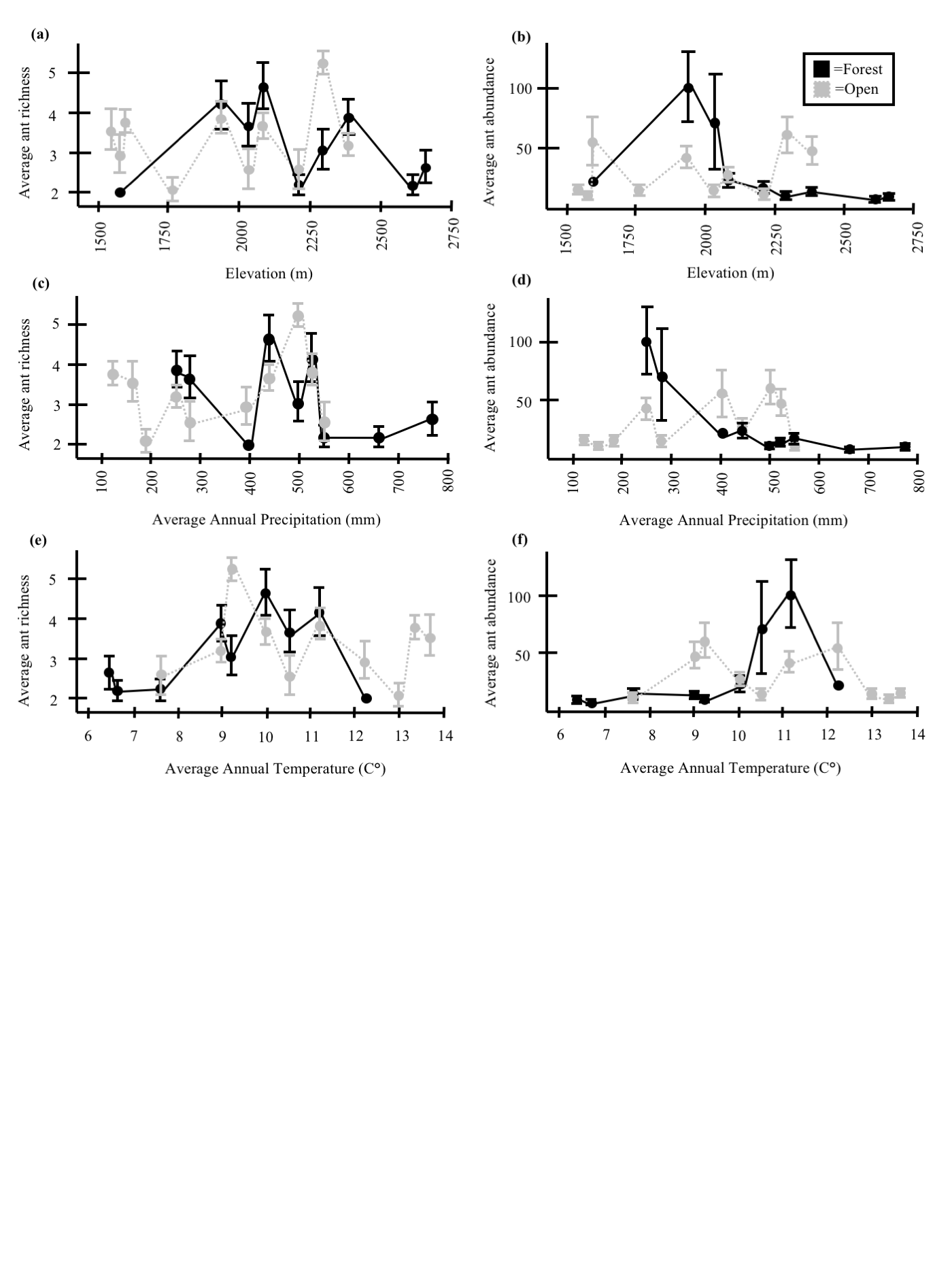


**Figure S3.** Average ant richness (a, c, and e) and abundance (b, d, and f) across elevation (a,b), average annual precipitation (c,d) and temperature (e,f).


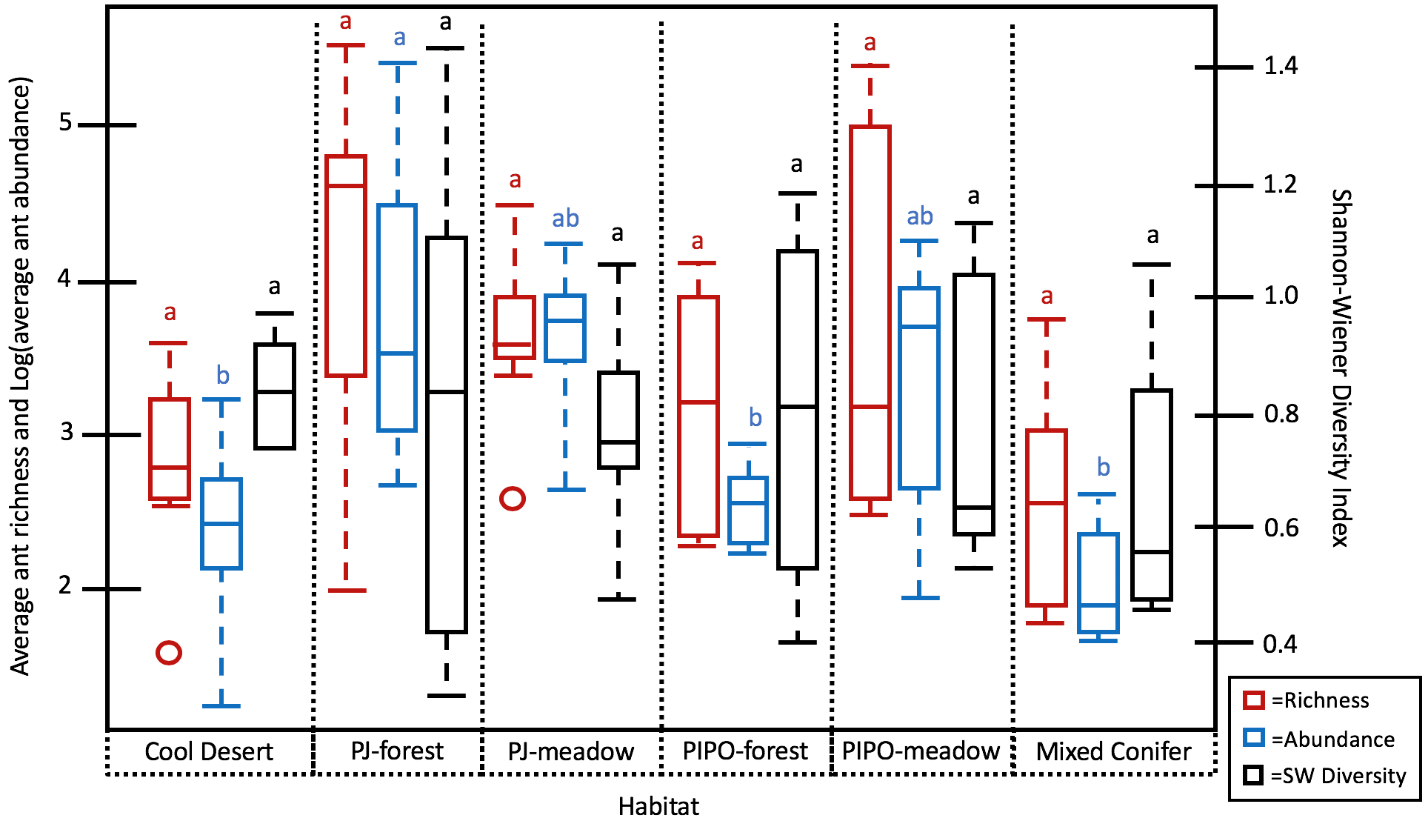


**Figure S4.** Box plot of ant richness, abundance, and Shannon-Wiener (SW) diversity index between habitat types in northern Arizona. Richness (*F*_5,29_=2.18, *p*=0.08) and SW diversity (*F*_5,29_=0.14, *p*=0.98) did not significantly differ between habitats, but abundance (*F*_5,29_=5.83, *p*<0.001) was significantly lower in mixed conifer, cool desert, and ponderosa meadows.

**Table S1.** Site descriptions for two elevational gradients sampled: C. Hart Merriam Elevational Gradient (C.H.M.) and Southwestern Experimental Garden Array (S.E.G.A.), 13 northern Arizona sites in total. Site characteristics consist of habitat (open desert, meadow, or forest), life zone (cool desert, pinyon/juniper, ponderosa, or mixed conifer), elevation (m), and 30 year averages of minimum/maximum temperature (^o^C). Also included is the number of plots sampled per each season at each site.

| Site name | Gradient | Habitat | Life Zone | Elevation (m) | # of June plots | # of August plots | Average min.-max temp./yr (^o^C) |
| --- | --- | --- | --- | --- | --- | --- | --- |
| Merriam 1 (MP1) | C.H.M. | Open desert | Cool desert | 1556 | 5 | 0 | 4-18 |
| Black Point (BP) | S.E.G.A. | Open desert | Cool desert | 1566 | 5 | 4 | 41-21 |
| Merriam 2 (MP2) | C.H.M. | Open desert | Cool desert | 1760 | 5 | 5 | 44-19 |
| Walnut Creek (WCF) | S.E.G.A. | Forest | Pinyon/juniper | 1567 | 1 | 0 | 3-22 |
| Walnut Creek (WCM) | S.E.G.A. | Meadow | Pinyon/juniper | 1567 | 5 | 5 | 3-22 |
| Merriam 3 (MP3F) | C.H.M. | Forest | Pinyon/juniper | 1930 | 5 | 0 | 4-19 |
| Merriam 3 (MP3M) | C.H.M. | Meadow | Pinyon/juniper | 1930 | 5 | 2 | 4-19 |
| Blue Chute (BCF) | S.E.G.A. | Forest | Pinyon/juniper | 2020 | 4 | 4 | 1-19 |
| Blue Chute (BCM) | S.E.G.A. | Meadow | Pinyon/juniper | 2020 | 5 | 5 | 1-19 |
| White Pocket (WPF) | S.E.G.A. | Forest | Pinyon/juniper | 2057 | 5 | 5 | 4-19 |
| White Pocket (WPM) | S.E.G.A. | Meadow | Pinyon/juniper | 2057 | 5 | 5 | 4-19 |
| Arboretum (AF) | S.E.G.A. | Forest | Ponderosa | 2179 | 5 | 4 | -1-16 |
| Arboretum (AM) | S.E.G.A. | Meadow | Ponderosa | 2200 | 5 | 4 | -1-16 |
| Little Mountain (LMF) | S.E.G.A. | Forest | Ponderosa | 2276 | 5 | 2 | 1-16 |
| Little Mountain (LMM) | S.E.G.A. | Meadow | Ponderosa | 2276 | 5 | 5 | 1-16 |
| Merriam 4 (MP4F) | C.H.M. | Forest | Ponderosa | 2344 | 5 | 5 | 3-19 |
| Merriam 4 (MP4M) | C.H.M. | Meadow | Ponderosa | 2344 | 5 | 5 | 3-19 |
| Merriam 5 (MP5) | C.H.M. | Forest | Mixed Conifer | 2620 | 5 | 10 | 0-13 |
| Bear Springs (BS) | S.E.G.A. | Forest | Mixed Conifer | 2688 | 4 | 5 | -1-14 |

**Table S2.** Voucher specimens of ants collected in northern Arizona, deposited at Northern Arizona University at the Colorado Plateau Museum of Arthropod Biodiversity and entered into the online data base Symbiota Collections of Arthropods Network (SCAN, [http://scan-bugs.org/portal/index.php]) and submitted to Bugguide.net for public access ( to access images, place the “bugguide number” into the end of the URL, www.bugguide.net/######). Identifications by James Trager and Ben Coulter were initially provided via images on bugguide.net, and later confirmed in-person by Gary Alpert.

| Taxonomic Identification | Authority | Determined by | Date Deter-mined | SCAN reference number | Bugguide reference number |
| --- | --- | --- | --- | --- | --- |
| *Camponotus hyatti* | Emery 1893 | Gary Alpert | 4/9/15 | NAUF4A0056895 | 1186094 |
| *Camponotus modoc* | Wheeler 1910 | Gary Alpert | 4/9/15 | NAUF4A0042166 | 1053715 |
| *Camponotus ocreatus* | Emery 1893 | Gary Alpert | 4/9/15 | NAUF4A0042167 | 1053725 |
| *Camponotus sansabeanus* | (Buckley 1866) | James Trager | 6/30/16 | NAUF4A0056768 | 1103574 |
| *Camponotus vicinus* | Mayr 1870 | James Trager | 3/6/15 | NAUF4A0042070 | 1043143 |
| *Crematogaster navajoa* | Buren 1968 | Gary Alpert | 10/20/15 | NAUF4A0056660 | 1115236 |
| *Crematogaster punctulata* | Emery 1895 | James Trager | 8/22/14 | NAUF4A0041915 | 982753 |
| *Dorymyrmex* (*smithi* complex) | Cole 1936 | Gary Alpert | 10/20/15 | NAUF4A0056591 | 1115254 |
| *Forelius mccooki* | McCook 1879 | Derek Uhey | 7/8/15 | NAUF4A0042095 | 1045881 |
| *Forelius pruinosus* | Roger 1863 | Derek Uhey | 7/8/15 | NAUF4A0042096 | 1045885 |
| *Formica aserva* | Forel 1901 | James Trager | 10/15/15 | NAUF4A0056641 | 1152200 |
| *Formica comata* | Wheeler 1912 | James Trager | 8/22/14 | NAUF4A0041914 | 982747 |
| *Formica criniventris* | Wheeler 1912 | Gary Alpert | 10/30/15 | Pending | 1167082 |
| *Formica densiventris* | Viereck 1903 | Ben Coulter | 11/20/15 | NAUF4A0041950 | 988953 |
| *Formica podzolica* | Francoeur 1973 | James Trager | 3/4/15 | NAUF4A0042083 | 1058992 |
| *Formica propinqua* | Creighton 1940 | James Trager |  | NAUF4A0056682 | 1115270 |
| *Formica* (*neogagates* group) | Viereck 1903 | James Trager | 11/18/15 | NAUF4A0041968 | 1163202 |
| *Formica subaenescens* | Emery 1893 | James Trager | 7/16/18 | NAUF4A0041932 | 985343 |
| *Lasius alienus* | (Foerster 1850) | Gary Alpert | 03/16 | Pending | Pending |
| *Lasius niger* | Wilson 1955 | James Trager | 3/6/15 | NAUF4A0042089 | 1119801 |
| *Lasius pallitarsis* | Provancher 1881 | James Trager | 10/10/14 | NAUF4A0041935 | 987031 |
| *Lasius sitiens* | Wilson 1955 | Gary Alpert | 10/30/15 | NAUF4A0042106 | 1059075 |
| *Lasius xerophilus* | Mackay & Mackay 1994 | Gary Alpert | 10/20/15 | NAUF4A0042192 | 1059058 |
| *Liometopum apiculatum* | Mayr 1870 | James Trager | 8/27/14 | NAUF4A0041933 | 985363 |
| *Monomorium cyaneum* | DuBois 1986 | Gary Alpert | 10/20/15 | NAUF4A0042093 | 1045872 |
| *Myrmecocystus (christineae)* | Snelling 1982 | Gary Alpert | 9/06/18 | NAUF4A0041946 | 988925 |
| *Myrmecocystus mexicanus* | Wesmael 1838 | Derek Uhey | 2/27/15 | NAUF4A0042069 | 1043142 |
| *Myrmecocystus mimicus* | Wheeler 1908 | Gary Alpert | 10/23/15 | NAUF4A0041916 | 982738 |
| *Myrmecocystus semirufus* | Emery 1893 | Gary Alpert | 10/23/15 | NAUF4A0041912 | 982738 |
| *Myrmica tahoensis* | Weber 1943 | Gary Alpert | 10/23/15 | NAUF4A0056777 | 1033157 |
| *Myrmica rugiventris* | Smith 1943 | Gary Alpert | 11/20/15 | NAUF4A0042185 | 1059011 |
| *Neivamyrmex nigrescens* | Cresson 1872 | James Trager | 8/22/14 | NAUF4A0041913 | 982742 |
| *Pheidole bicarinata* | Creighton 1950 | Gary Alpert | 10/20/15 | NAUF4A0042068 | 1043129 |
| *Pheidole ceres* | Wheeler 1904 | Gary Alpert | 10/20/15 | NAUF4A0042188 | 1103063 |
| *Pheidole senex* | Greg 1952 | Gary Alpert | 10/20/15 | NAUF4A0042097 | 1059066 |
| *Pheidole* morphospecies |  |  |  | Pending | 1582338 |
| *Pogonomyrmex californicus* | (Buckley 1867) | Gary Alpert | 3/6/15 | NAUF4A0041929 | Pending |
| *Pogonomyrmex huachucanus* | Wheeler 1914 | Derek Uhey | 8/20/14 | NAUF4A0041910 | 982304 |
| *Pogonomyrmex rugosus* | Emery 1895 | James Trager | 3/5/15 | NAUF4A0042066 | 985328 |
| *Pogonomyrmex occidentalis* | (Cresson 1865) | Derek Uhey | 12/10/14 | NAUF4A0041931 | 985336 |
| *Stigmatomma pallipes* | (Halderman 1844) | James Trager | 3/01/15 | NAUF4A0042065 | 1043116 |
| *Strumigenys* | morpho-species | James Trager, Douglas Booher | 03/15 | NAUF4A0042088 | 1044580 |
| *Tapinoma sessile* | Creighton 1950 | Gary Alpert | 10/23/15 | Pending | Pending |
| *Temnothorax tricarinatus* | Emery 1895 | Gary Alpert | 10/23/15 | NAUF4A0042153 | 1051434 |

**Table S3.** Weather and climate data for elevational gradient sites. Climate data consisted of 30-year averages of temperature and precipitation taken from PRISM climate group (<http://prism.oregonstate.edu>). Weather data was collected via on-site weather stations, temperature data-loggers, or from nearby weather stations at equivalent elevations. Correlations of weather and climate variables with each other, and with elevation, are given, with significant correlations bolded.

| Site name | PRISM data | | Weather Data | | | | Elevation (m) |
| --- | --- | --- | --- | --- | --- | --- | --- |
|  | Average annual precip. (mm) | Average annual temp.  (^o^C) | June Precip.  (mm) | Aug Precip.  (mm) | June Average Temp. | Aug Average Temp. |  |
| Merriam Great Basin | 127 | 13.6 | 0 ^A^ | 0 ^A^ | 21.8 ^A^ | 22.6 ^A^ | 1556 |
| Black Point | 152 | 13.37 | 0 ^A^ | 0 ^A^ | 22.6 ^B^ | 21.4 ^B^ | 1566 |
| Merriam Grassland | 178 | 11.76 | 16 ^A^ | 60 ^A^ | 20.5 ^A^ | 19.9 ^A^ | 1760 |
| Walnut Creek | 397 | 12.16 | 0 ^B^ | 0 ^B^ | 21.8 ^B^ | 16 ^B^ | 1567 |
| Blue Chute | 478 | 9.69 | 0 ^B^ | 148 ^A^ | 18.5 ^B^ | 25 ^A^ | 1930 |
| Merriam Pinyon/Juniper | 254 | 11.09 | 2 ^A^ | 43.2 ^A^ | 18.6 ^A^ | 19.8 ^A^ | 2020 |
| White Pocket | 443 | 10.03 | 0 ^B^ | 151 ^A^ | 17.6 ^B^ | 22 ^A^ | 2057 |
| Arboretum | 556 | 7.64 | 0 ^B^ | 76.6 ^A^ | 15.5 ^B^ | 23.1 ^A^ | 2200 |
| Little Mountain | 502 | 9.12 | 0 ^B^ | 30.5 ^E^ | 15.4 ^B^ | 16.9 ^B^ | 2276 |
| Merriam Ponderosa | 356 | 10.13 | 7.9 ^A^ | 36.6 ^A^ | 16.5 ^A^ | 19.5 ^A^ | 2344 |
| Merriam Mixed Conifer | 508 | 10.34 | 0.2 ^B^ | 41.4 ^B^ | 13.6 ^B^ | 14.8 ^B^ | 2620 |
| Bear Springs | 772 | 6.36 | 0 ^B^ | 30.5 ^B^ | 12.4 ^B^ | 16.9 ^B^ | 2688 |
| *Correlations of elevation, climate, and weather variables* | | | | | | | |
| Average annual precip. | -- |  |  |  |  |  | **0.78** |
| Average annual temp. | **-0.92** | -- |  |  |  |  | **-0.82** |
| June Precip | **-0.39** | 0.17 | -- |  |  |  | -0.10 |
| Aug Precip. | **0.29** | **-0.38** | 0.00 | -- |  |  | 0.17 |
| June Average Temp. | **-0.85** | **0.88** | 0.17 | -0.23 | -- |  | **-0.98** |
| Aug Average Temp. | **-0.31** | 0.13 | -0.014 | **0.53** | **0.38** | -- | **-0.43** |

^A^ = Measurement from weather station on site

^B^ = Measurement from temperature data-logger

**Table S4.** Taxonomic list of ants with averages and standard errors per life zone/habitat combination along elevational gradients in northern Arizona. Specimens were identified to species level when possible. The few groups not identified to species were treated as single units for conservative estimates when comparing groups. Bolded occurrences denote significant indicator values for that species in that group (p<0.05, Supp. Table 6). Correlations with two axes of a NMDS ordination based on site composition are given.

| Subfamily | Taxonomic identity and authority | **Cool Desert**  sites=3, pit traps=24 | **Pinyon/juniper-meadow**  Sites=4, pit traps=37 | **Pinyon/juniper-forest**  Sites=4, pit traps=24 | **Ponderosa-meadow**  Sites=3, pit traps=29 | **Ponderosa-forest**  Sites=3, pit traps=26 | **Mixed conifer**  Sites=3, pit traps=24 | **Correlation (r) with NMDS axis one** | **Correlation (r) with NMDS axis two** |
| --- | --- | --- | --- | --- | --- | --- | --- | --- | --- |
| Dorylinae | *Neivamyrmex nigrescens* |  | 0.09 (0.06) |  |  |  |  | 0.02 | 0.01 |
|  | Cresson 1872 |  |  |  |  |  |  |  |  |
| Dolichoderinae | *Liometopum apiculatum* |  | **1.00 (0.61**) | 0.82 (0.53) |  |  |  | -0.17 | -0.43 |
|  | Mayr 1870 |  |  |  |  |  |  |  |  |
|  | *Tapinoma sessile* |  |  |  | **6.58 (4.58)** | 3.13 (1.57) | 0.92 (0.46) | **0.30** | -0.01 |
|  | Creighton 1950 |  |  |  |  |  |  |  |  |
|  | *Dorymrymex* (*insanus* complex) | 1.33 (0.64) | 12.65 (6.26) | 45.5 (23.48) | 0.08 (0.08) | 0.69 (0.41) |  | **-0.32** | **-0.31** |
|  | Cole 1936 |  |  |  |  |  |  |  |  |
|  | *Forelius mccooki* | 1.00 (0.45) | 1.00 (0.37) | 0.04 (0.04) |  |  |  | **-0.31** | 0.13 |
|  | McCook 1879 |  |  |  |  |  |  |  |  |
|  | *Forelius pruinosus* | 3.58 (1.68) | **7.76 (3.04)** | 4.18 (1.64) |  |  |  | **-0.49** | -0.04 |
|  | Roger 1863 |  |  |  |  |  |  |  |  |
| Formicinae | *Lasius niger* |  |  |  | **9.23 (3.52)** | 1.45 (0.63) |  | 0.15 | -0.13 |
|  | Wilson 1955 |  |  |  |  |  |  |  |  |
|  | *Lasius sitiens* |  | 0.03 (0.03) | 0.50 (0.34) | 1.08 (0.29) | **2.97 (1.77)** |  | 0.17 | 0.09 |
|  | Wilson 1955 |  |  |  |  |  |  |  |  |
|  | *Myrmecocystus mexicanus* | 0.42 (0.20) |  |  |  |  |  | -0.16 | 0.20 |
|  | Wesmael 1838 |  |  |  |  |  |  |  |  |
|  | *Myrmecocystus mimicus* | **0.17 (0.08)** |  |  |  |  |  | -0.18 | 0.45 |
|  | Wheeler 1908 |  |  |  |  |  |  |  |  |
|  | *Myrmecocystus semirufus* |  | 0.03 (0.03) |  |  |  |  | 0.20 | 0.09 |
|  | Emery 1893 |  |  |  |  |  |  |  |  |
|  | *Formica aserva* |  |  |  |  |  | **1.50 (0.81)** | 0.16 | **0.42** |
|  | Forel 1901 |  |  |  |  |  |  |  |  |
|  | *Formica comata* |  |  |  | 0.04 (0.04) | 0.08 (0.05) |  | 0.32 | -0.07 |
|  | Wheeler 1909 |  |  |  |  |  |  |  |  |
|  | *Formica criniventris* |  |  |  | 2.50 (1.2) | 1.17 (0.81) |  | 0.09 | -0.18 |
|  | Wheeler 1912 |  |  |  |  |  |  |  |  |
|  | *Formica (neogagates* complex*)* |  | 1.85 (0.79) | 0.18 (0.12) | 2.15 (1.72) | 0.38 (0.16) |  | 0.66 | **0.31** |
|  | Viereck 1903 |  |  |  |  |  |  |  |  |
|  | *Formica podzolica* |  |  | 0.04 (0.04) | 0.38 (0.27) | 0.28 (0.17) | **2.08 (0.54)** | 0.02 | 0.10 |
|  | Francoeur 1973 |  |  |  |  |  |  |  |  |
|  | *Camponotus hyatti* |  |  | 0.07 (0.07) |  |  |  | 0.08 | 0.09 |
|  | Emery 1893 |  |  |  |  |  |  |  |  |
|  | *Camponotus modoc* |  |  |  |  |  | **0.54 (0.15)** | **-**0.26 | **-0.44** |
|  | Wheeler 1910 |  |  |  |  |  |  |  |  |
|  | *Camponotus ocreatus* |  | 0.06 (0.04) | **0.86 (0.23)** |  |  |  | -0.20 | -0.15 |
|  | Emery 1893 |  |  |  |  |  |  |  |  |
|  | *Camponotus vicinus* |  | 0.09 (0.05) | 0.89 (0.25) |  | 0.59 (0.19) | 0.04 (0.04) | 0.53 | 0.04 |
|  | Mayr 1870 |  |  |  |  |  |  |  |  |
| Myrmicinae | *Myrmica* spp. |  |  |  | 0.04 (0.04) | 0.38 (0.18) | **2.58 (1.26)** | 0.55 | 0.22 |
|  | Creighton 1950 |  |  |  |  |  |  |  |  |
|  | *Myrmica rugiventris* |  |  | 0.04 (0.04) |  |  |  | -0.15 | -0.18 |
|  | Smith 1943 |  |  |  |  |  |  |  |  |
|  | *Myrmica tahoensis* |  |  | 0.25 (0.13) |  |  |  | 0.02 | -0.09 |
|  | Weber 1948 |  |  |  |  |  |  |  |  |
|  | *Pogonomyrmex californicus* | 0.04 (0.04) |  |  |  |  |  | 0.09 | 0.20 |
|  | (Buckley 1867) |  |  |  |  |  |  |  |  |
|  | *Pogonomyrmex huachucanus* |  |  | 0.11 (0.06) |  |  |  | 0.03 | 0.09 |
|  | Wheeler 1914 |  |  |  |  |  |  |  |  |
|  | *Pogonomyrmex occidentalis* |  | 0.29 (0.16) | 0.04 (0.04) |  |  |  | -0.25 | -0.09 |
|  | (Cresson 1865) |  |  |  |  |  |  |  |  |
|  | *Pogonomyrmex rugosus* | **0.625 (0.25)** |  |  |  |  |  | -0.29 | **0.38** |
|  | Emery 1895 |  |  |  |  |  |  |  |  |
|  | *Solenopsis* (*fugax* group) | 0.375 (0.26) | 0.32 (0.13) | 0.18 (0.08) | 0.92 (0.23) | 0.48 (0.18) |  | 0.181 | 0.04 |
|  | Westwood 1840 |  |  |  |  |  |  |  |  |
|  | *Monomorium cyaneum* | 0.125 (0.09) | 5.29 (2.86) | 1.61 (0.74) | 3.08 (1.71) | 0.38 (0.18) | 0.04 (0.04) | 0.03 | **-0.30** |
|  | DuBois 1986 |  |  |  |  |  |  |  |  |
|  | *Pheidole* sp. (morpho-species)  Westwood1839 |  |  |  | 8.42 (4.14) | 0.72 (0.49) |  | 0.15 | **0.37** |
|  | *Pheidole bicarinata* | **4.83 (1.04)** |  |  |  |  |  | **-0.35** | **0.45** |
|  | Creighton 1950 |  |  |  |  |  |  |  |  |
|  | *Pheidole ceres* |  | **5.82 (2.21)** | 5.04 (2.67) |  |  |  | **-0.43** | **-0.35** |
|  | Wheeler 1904 |  |  |  |  |  |  |  |  |
|  | *Pheidole senex* |  | 0.03 (0.03) | 0.04 (0.04) | **3.81 (2.53)** | 0.10 (0.08) |  | 0.12 | -0.23 |
|  | Greg 1952 |  |  |  |  |  |  |  |  |
|  | *Temnothorax tricarinatus* |  |  |  | 0.12 (0.12) |  |  | 0.11 | 0.02 |
|  | Emery 1895 |  |  |  |  |  |  |  |  |
|  | *Crematogaster navajoa* |  | 0.21 (0.11) | 1.07 (0.53) | 0.03 (0.03) |  |  | -0.13 | -0.02 |
|  | Buren 1968 |  |  |  |  |  |  |  |  |
|  | *Crematogaster punctulata* |  | 1.20 (0.77) | 15.75 (11.14) |  |  |  | -0.15 | **-0.42** |
|  | Emery 1895 |  |  |  |  |  |  |  |  |
| Richness (note uneven sampling) | | 10 | 17 | 20 | 15 | 14 | 7 |  |  |
| Total indicator species | | 3 | 3 | 1 | 3 | 1 | 4 |  |  |

**Table S5.** Results of pairwise perMANOVA comparing ant communities in life zone/habitat combinations.

| Life Zones/Habitat | Df | Pseudo-F | R^2^ | adjusted p-value |
| --- | --- | --- | --- | --- |
| Cool Desert vs Pinyon/Juniper forest | 49 | 7.7562 | 0.1391 | 0.015 |
| Cool Desert vs Pinyon/Juniper meadow | 56 | 9.1723 | 0.1429 | 0.015 |
| Cool Desert vs Ponderosa forest | 51 | 10.5206 | 0.1738 | 0.015 |
| Cool Desert vs Ponderosa meadow | 48 | 10.4439 | 0.1818 | 0.015 |
| Cool Desert vs Mixed Conifer | 46 | 16.2619 | 0.2654 | 0.015 |
| Pinyon/Juniper forest vs Pinyon/Juniper meadow | 60 | 1.7675 | 0.0290 | 0.63 |
| Pinyon/Juniper forest vs Ponderosa forest | 55 | 5.1407 | 0.0869 | 0.015 |
| Pinyon/Juniper forest vs Ponderosa meadow | 52 | 5.9191 | 0.1039 | 0.015 |
| Pinyon/Juniper forest vs Mixed Conifer | 50 | 11.0752 | 0.1843 | 0.015 |
| Pinyon/Juniper meadow vs Ponderosa forest | 62 | 7.8954 | 0.1146 | 0.015 |
| Pinyon/Juniper meadow vs Ponderosa meadow | 59 | 7.4504 | 0.1138 | 0.015 |
| Pinyon/Juniper meadow vs Mixed Conifer | 57 | 13.8108 | 0.1978 | 0.015 |
| Ponderosa forest vs Ponderosa meadow | 54 | 2.2494 | 0.0407 | 0.06 |
| Ponderosa forest vs Mixed Conifer | 52 | 7.1594 | 0.1231 | 0.015 |
| Ponderosa meadow vs Mixed Conifer | 49 | 9.5900 | 0.1665 | 0.015 |

**Supplementary Table 6.** Indicator values, average abundance, standard error, and p-value (obtained from Monte Carlo) of ant taxa by life zone.

| Ant taxa | Max Life Zone | Observed Indicator Value | Average | Standard Error | p-value |
| --- | --- | --- | --- | --- | --- |
| *Camponotus modoc* | Mix Conifer | 80 | 14.5 | 8.4 | 0.0002 |
| *Camponotus ocreatus* | Pinyon/Juniper | 50 | 18.2 | 9.2 | 0.0072 |
| *Camponotus vicinus* | Pinyon/Juniper | 32.5 | 22.9 | 8.98 | 0.1362 |
| *Crematogaster navajoa* | Pinyon/Juniper | 35 | 18.5 | 9.84 | 0.0576 |
| *Crematogaster punctulata* | Pinyon/Juniper | 21.4 | 15.3 | 8.52 | 0.1406 |
| *Dorymrymex (smithi* complex*)* | Pinyon/Juniper | 60.2 | 32.9 | 12.54 | 0.0406 |
| *Forelius mccooki* | Cool Desert | 41 | 16.9 | 9.04 | 0.0278 |
| *Forelius pruinosus* | Pinyon/Juniper | 64.5 | 27.2 | 10.31 | 0.0048 |
| *Formica (criniventris)* | Ponderosa | 33.3 | 14.8 | 8.39 | 0.0338 |
| *Formica aserva* | Mix Conifer | 40 | 12.9 | 6.76 | 0.0344 |
| *Formica (neogatates* complex*)* | Ponderosa | 22.7 | 21.1 | 10.09 | 0.3449 |
| *Formica podzolica* | Mix Conifer | 69.6 | 19 | 9.23 | 0.0004 |
| *Lasius niger* | Ponderosa | 66.7 | 21.3 | 10.3 | 0.0034 |
| *Lasius sitiens* | Ponderosa | 68.2 | 24.6 | 10.69 | 0.0022 |
| *Liometopum apiculatum* | Pinyon/Juniper | 35.7 | 17.1 | 9.12 | 0.047 |
| *Monomorium cyaneum* | Pinyon/Juniper | 40.2 | 32.6 | 11.07 | 0.2116 |
| *Myrmecocystus mexicanus* | Cool Desert | 20 | 11 | 5.5 | 0.2721 |
| *Mrymecocystus mimicus* | Cool Desert | 60 | 13.9 | 7.48 | 0.0026 |
| *Myrmica* | Mix Conifer | 91.8 | 22.8 | 10.54 | 0.0004 |
| *Myrmica tahoensis* | Pinyon/Juniper | 21.4 | 13.9 | 7.61 | 0.1796 |
| *Pheidole* #1 MP4 | Ponderosa | 33.3 | 16.2 | 8.61 | 0.0482 |
| *Pheidole bicarinata* | Cool Desert | 100 | 15.8 | 8.83 | 0.0002 |
| *Pheidole ceres* | Pinyon/Juniper | 78.6 | 22.4 | 9.82 | 0.0008 |
| *Pheidole senex* | Ponderosa | 48.7 | 23.5 | 11.5 | 0.051 |
| *Pogonomyrmex occidentalis* | Pinyon/Juniper | 28.6 | 15.5 | 8.21 | 0.1068 |
| *Pogonomyrmex rugosus* | Cool Desert | 100 | 16.5 | 8.82 | 0.0002 |
| *Solenopsis (fugax* group*)* | Ponderosa | 47.6 | 25.8 | 8.04 | 0.0208 |
| *Tapinoma sessile* | Ponderosa | 63.2 | 27.3 | 10.99 | 0.0106 |
